# Exogenous Melatonin Mitigates Photoinhibition by Accelerating Non-photochemical Quenching in Tomato Seedlings Exposed to Moderate Light during Chilling

**DOI:** 10.3389/fpls.2017.00244

**Published:** 2017-02-20

**Authors:** Fei Ding, Meiling Wang, Bin Liu, Shuoxin Zhang

**Affiliations:** ^1^College of Forestry, Northwest A&F UniversityYangling, China; ^2^College of Agronomy, Northwest A&F UniversityYangling, China

**Keywords:** chilling, melatonin, non-photochemical quenching, photoinhibition, *Solanum lycopersicum*, violaxanthin de-epoxidase, xanthophyll cycle

## Abstract

Melatonin plays an important role in tolerance to multiple stresses in plants. Recent studies have shown that melatonin relieves photoinhibition in plants under cold stress; however, the mechanisms are not fully understood. Non-photochemical quenching (NPQ) is a key process thermally dissipating excess light energy that plants employ as a protective mechanism to prevent the over reduction of photosystem II. Here, we report the effects of exogenous melatonin on NPQ and mitigation of photoinhibition in tomato seedlings exposed to moderate light during chilling. In response to moderate light during chilling, the maximum quantum yield (Fv/Fm) and the effective photochemical efficiency (F′v/F′m) of PSII were both substantially reduced, showing severe photoinhibition in tomato seedlings, whereas exogenous application of melatonin effectively alleviated the photoinhibition. Further experiment showed that melatonin accelerated the induction of NPQ in response to moderate light and maintained higher level of NPQ upon longer exposure to light during chilling. Consistent with the increased NPQ was the elevated de-epoxidation state of xanthophyll pigments in melatonin-pretreated seedlings exposed to light during chilling. Enzyme activity assay showed that violaxanthin de-epoxidase (VDE), which catalyzes the de-epoxidation reaction in the xanthophyll cycle, was activated by light and the activity was further enhanced by application of melatonin. Further analysis revealed that melatonin induced the expression of VDE gene in tomato seedlings under moderate light and chilling conditions. Ascorbic acid is an essential cofactor of VDE and the level of it was found to be increased in melatonin-pretreated seedlings. Feeding tomato seedlings with dithiothreitol, an inhibitor of VDE, blocked the effects of melatonin on the de-epoxidation state of xanthophyll pigments and the induction of NPQ. Collectively, these results suggest that exogenous melatonin mitigates photoinhibition by accelerating NPQ through the stimulation of VDE activity and the enhancement of de-epoxidation state of xanthophyll pigments.

## Introduction

Plants are largely dependent on the efficient conversion of absorbed light energy to chemical energy to sustain growth and development. However, under high light, light absorption generally exceeds photochemical demands in plants, inevitably leading to energy imbalance (Björkman and Demmig-Adams, 1994; Kaňa and Govindjee, 2016; Zhao et al., 2017). The resulting energy imbalance can be exacerbated by environmental stresses, such as drought, high or low temperatures, and salinity (Takahashi and Murata, 2008). The excess light energy can ultimately result in the generation of destructive singlet oxygen and other reactive oxygen species (ROS) (Niyogi, 1999), which pose severe oxidative damage to photosynthetic apparatus (Melis, 1999; Yin et al., 2010). Not surprisingly, in the long-term evolution, plants have developed multiple mechanisms to balance excess light absorption with photochemical utilization in order to protect photosystems against photodamages (Horton et al., 1996; Niyogi et al., 2001). One important mechanism is to thermally dissipate excess absorbed light energy in the light-harvesting antenna complexes of photosystem II (PSII), which confers protection of PSII against inactivation and potential damages by excess light energy. This process of thermally dissipation is referred to as non-photochemical quenching (NPQ). NPQ involves energy-dependent quenching (qE), quenching associated with state transition (qT) and photoinhibition (qI), among which qE acts predominantly to dissipate excess excitation energy absorbed in the PSII antenna as heat and thus plays an important role in alleviation of PSII photoinhibition (Wraight and Crofts, 1970; Briantais et al., 1979; Horton et al., 1996; Nilkens et al., 2010).

The qE component of NPQ relies on the light-mediated de-epoxidation of violaxanthin to zeaxanthin in the xanthophyll cycle, which requires acidification of thylakoid lumen (Niyogi et al., 1998; Munekage et al., 2001). In the xanthophyll cycle, the de-epoxidation of violaxanthin to antheraxanthin and zeaxanthin is catalyzed by violaxanthin de-epoxidase (VDE). VDE is a 43 kD protein encoded by the nuclear gene *VDE*/*NPQ1* and its activation requires acidification of thylakoid lumen as a result of light-driven electron movement through the photosynthetic electron transport chain (Briantais et al., 1979; Pfündel and Dilley, 1993; Hager and Holocher, 1994). VDE activity is also influenced by ascorbic acid, which is an essential cofactor of VDE (Bratt et al., 1995; Smirnoff, 1996, 2000a,b). Suppression of dehydroascorbate reductase (DHAR) expression, which is responsible for the generation of ascorbic acid, results in reductions in xanthophyll pigments, reduced NPQ and increased photoinhibition (Chen and Gallie, 2008). While VDE activity is light-dependent, *VDE* transcript expression in *Arabidopsis* is suppressed by light and induced by drought under light (North et al., 2005). Moreover, in most cases transcriptional regulation of VDE gene is not correlated to protein level and activity (Bugos et al., 1999).

Melatonin (*N*-acetyl-5-methoxytryptamine) is an important hormone involved a number of biological processes in animals. Recently, melatonin has also been demonstrated to play important roles in plants. As an indoleamine, melatonin functions as an auxin-like hormone regulating root development in plants (Murch et al., 2001; Zhang et al., 2014). Melatonin is also involved in the delay of leaf senescence (Wang et al., 2013; Shi et al., 2015b). Moreover, melatonin mitigates oxidative stress by directly scavenging ROS or indirectly improving antioxidant potential (Arnao and Hernández-Ruiz, 2015; Reiter et al., 2015). Studies have also shown that melatonin confers tolerance to a variety of abiotic and biotic stresses in plants, including cold, heat, salinity, drought, heavy metal toxicity, and pathogens (Li et al., 2012, 2016; Bajwa et al., 2014; Shi et al., 2014, 2015a; Xu et al., 2016). Recent studies demonstrate that melatonin alleviates damages to photosystems induced by cold and salinity through enhancement of antioxidant capacity and regulation of electron transport chain (Fan et al., 2015; Szafrańska et al., 2016; Zhao et al., 2016; Zhou et al., 2016). In unicellular organisms, melatonin may also play important roles. In a study on cultured *Symbiodinium*, melatonin was found to stimulate xanthophyll cycle activity and increase NPQ levels as a protective mechanism against excess solar energy (Roopin et al., 2013). In the last decade, significant progress has been made in deciphering the function of melatonin in stress responses in plants; however, the role of melatonin in the alleviation of photoinhibition is only partially understood and merits further investigation.

Tomato (*Solanum lycopersicum* L.) is an important horticultural crop worldwide; however, it is highly sensitive to low temperatures because of its tropical origin. Low temperatures, particularly under light, adversely affect all aspects of tomato plants including photosynthesis, and cause severe reductions in tomato yields (Park et al., 2004; Zushi et al., 2012; Ding et al., 2016, 2017). Thus exploring melatonin-mediated alleviation of photoinhibition in tomato is of both theoretical and practical significance. The objectives of this work were to investigate the role of melatonin in regulating NPQ in tomato seedlings exposed to moderate light during chilling and thus to explore the role of melatonin in relieving photoinhibition.

## Materials and Methods

### Plant Materials, Growth Conditions, and Treatment

Tomato (*Solanum lycopersicum* L. cv. Micro-Tom) seeds were sterilized and germinated at 25°C in the dark on filter paper in Petri dishes. Germinated seeds were then planted in 12 cm × 12 cm plastic pots containing peat and vermiculite (3/1, v/v) and maintained in a growth room with the following conditions: 380 ppm of CO_2_, photon flux density of 400 μmol m^-2^ s^-1^, day/night temperature of 25/20°C, relative humidity of 60% and a photoperiod of 14 h.

After the third leaf emerged, tomato seedlings were sprayed one time a day either with 100 μM melatonin (Sigma-Aldrich, St. Louis, MO, USA) solution or with distilled water for 3 days, which gave rise to two groups of seedlings. Then seedlings in each group were randomly divided into two subgroups. At the end of light cycle at 20:00 on day 3, one subgroup of each group was subjected to cold stress (4°C) in the dark and the rest of subgroups were kept under 25°C in the dark, then next morning at 6:00, all groups were exposed to light, resulting in four different subgroups: (1) Control: seedlings grown under 25°C first in the dark, then in the light (400 μmol m^-2^ s^-1^) at 6:00 next morning; (2) Control + MT: seedlings pretreated with melatonin and grown under 25°C first in the dark, then in the light (400 μmol m^-2^ s^-1^) at 6:00 next morning; (3) Chilling: seedlings exposed to 4°C first in the dark, then in the light (400 μmol m^-2^ s^-1^) at 6:00 next morning; (4) Chilling + MT: seedlings pretreated with melatonin exposed to 4°C first in the dark, then in the light (400 μmol m^-2^ s^-1^) at 6:00 next morning. For each treatment, there were a total of 45 tomato seedlings and each of three replicates consisted of 15 tomato seedlings.

Leaf samples from four subgroups were collected at 0, 5, 10, 30, and 60 min following exposure to light next morning and then immediately placed in liquid nitrogen. Then, samples were stored at -80°C for further analysis. Chlorophyll fluorescence was recorded at 20 s intervals for the initial 180 s and then every 30 min for 6 h following illumination.

### Measurement of Chlorophyll Fluorescence

Chlorophyll fluorescence was measured with a portable fluorometer (PAM-2000, Walz, Germany). The effective photochemical efficiency (F′v/F′m) and the maximum quantum efficiency (Fv/Fm) of PSII were measured in light-adapted seedlings and dark-adapted seedlings, respectively. The initial chlorophyll fluorescence yield (Fo) was determined under low-modulated light, followed by a pulse of saturating white light to obtain maximum fluorescence yield (Fm) in seedlings in the dark. The steady-state fluorescence levels (Fs) and the maximum fluorescence levels (Fm′) were monitored at different time points during light exposure. NPQ was estimated from the Stern–Volmer equation as: (Fm–F′m)/F′m. The specific procedures were followed as described by Chen and Gallie (2012).

### Analyses of Pigments in the Xanthophyll Cycle

Analyses of pigments in the xanthophyll cycle were performed as described by Thayer and Björkman (1990). Leaf samples were homogenized in 100% cold acetone and pigments extracts were filtered, then xanthophyll pigments were separated and quantified by HPLC.

### Determination of Transcript Abundance by Quantitative Real-Time PCR

Total RNA was extracted from seedling leaves and was used for cDNA synthesis by PrimeScript^®^ reverse transcriptase following standard protocols. Quantitative real-time PCR was performed using SYBR^®^ Premix Ex TaqTM (TaKaRa) according to manufacturer’s instructions. Each real-time PCR reaction was performed in 25 μl final volume on iQ5 Multicolor Real-Time PCR Detection System (Bio-Rad, USA) under the following program: 1 cycle of 30 s at 95°C, followed by 40 cycles of 5 s at 95°C and 30 s at 60°C. The primers for tomato *VDE* were AGTGCAGGATAGAGCTTGCG (Forward) and CGGGAGACTGCACACTCATT (Reverse). The primers for tomato *DHAR* were CTTCGAGCGAGAGTCGTTCC (Forward) and TAAAGCTGCACTCGTCGAACT (Reverse).

### Isolation of Chloroplasts

Chloroplasts were isolated as described in a previous study (Robinson et al., 1983). Ten grams of seedling leaves were extracted with buffer containing 330 mM sorbitol, 30 mM Mes, 2 mM ascorbate and 0.1% BSA. The crude extract was filtered and centrifuged at 1200 ×*g* for 3 min. The resulting pellets were re-suspended in buffer containing 330 mM sorbitol, 30 mM Hepes, and 0.2% BSA. The suspension was mixed with 80% percoll and 40% percoll, and was centrifuged at 1200 ×*g* for 1 min. The intact chloroplasts were isolated between 80% percoll and 40% percoll.

### VDE Activity Assay

Violaxanthin de-epoxidase activity was measured as previously described (Bugos et al., 1999; Chen and Gallie, 2012). Briefly, VDE activity was assayed in a reaction mixture containing 10 μL of 1 μM violaxanthin, 25 μL of 300 μM monogalactosyldiacylglycerol in methanol, 550 μL of 0.2 M sodium citrate (pH 5.1), and 50 μL of VDE extract. The reaction mixture was thoroughly mixed and incubated at 30°C for 5 min. The reaction was started by adding 6 μL of 3 M sodium ascorbate. After 10 min, the reaction was stopped by the addition of 1 N NaOH. The mixture was centrifuged at 20,000 ×*g* for 2 min and the resulting pellets containing the lipids and pigments were analyzed by HPLC.

### Dehydroascorbate Reductase (DHAR) Activity Assay

Dehydroascorbate reductase activity was analyzed essentially following Dalton et al. (1986). Crude enzyme extract was obtained by homogenizing a total volume of 3 mL of chloroplast suspension with 25 mM cold Hepes buffer (pH 7.8) containing 0.2 mM EDTA and 2% PVP. Following centrifugation at 4°C at 13, 000 ×*g* for 10 min, the supernatant was used to measure DHAR activity. One hundred μL enzyme extract was added to the reaction mixture containing 100 mM Hepes (pH 7.0), 1 mM EDTA, and 2.5 mM reduced glutathione. The reaction was initiated by adding 0.2 mM dehydroascorbate to reaction mixture and the increase in absorbance at 265 nm was measured as ascorbic acid was formed.

### Determination of Ascorbic Acid

A volume of 600 μL chloroplast suspension was homogenized in 1.2 mL of 6% (v/v) cold HClO_4_ and centrifuged at 4°C for 10 min at 10,000 *g*. The supernatant was used to determine the level of ascorbic acid as previously described (Logan et al., 1998). Ascorbic acid was assayed by determining the absorbance difference of the supernatant at 265 nm in 200 mM sodium acetate buffer (pH 5.6) before and after 15-min incubation with 1.5 units of ascorbate oxidase.

### Dithiothreitol (DTT) Feeding

Dithiothreitol (DTT) feeding experiment was carried out in tomato seedlings pretreated with or without melatonin under chilling stress. Tomato seedlings were infiltrated with either 5 mM DTT or with water via petiole 3 h before they were exposed to light.

### Statistical Analysis

All experiments in the present study were repeated at least three times, and the values presented are mean ± SD. Duncan’s multiple range test was performed to compare the difference among treatments. Different letters in figures indicate significant differences at *P* < 0.05.

## Results

### Melatonin Relieves Photoinhibition in Tomato Seedlings Exposed to Moderate Light during Chilling

**FIGURE 1:**
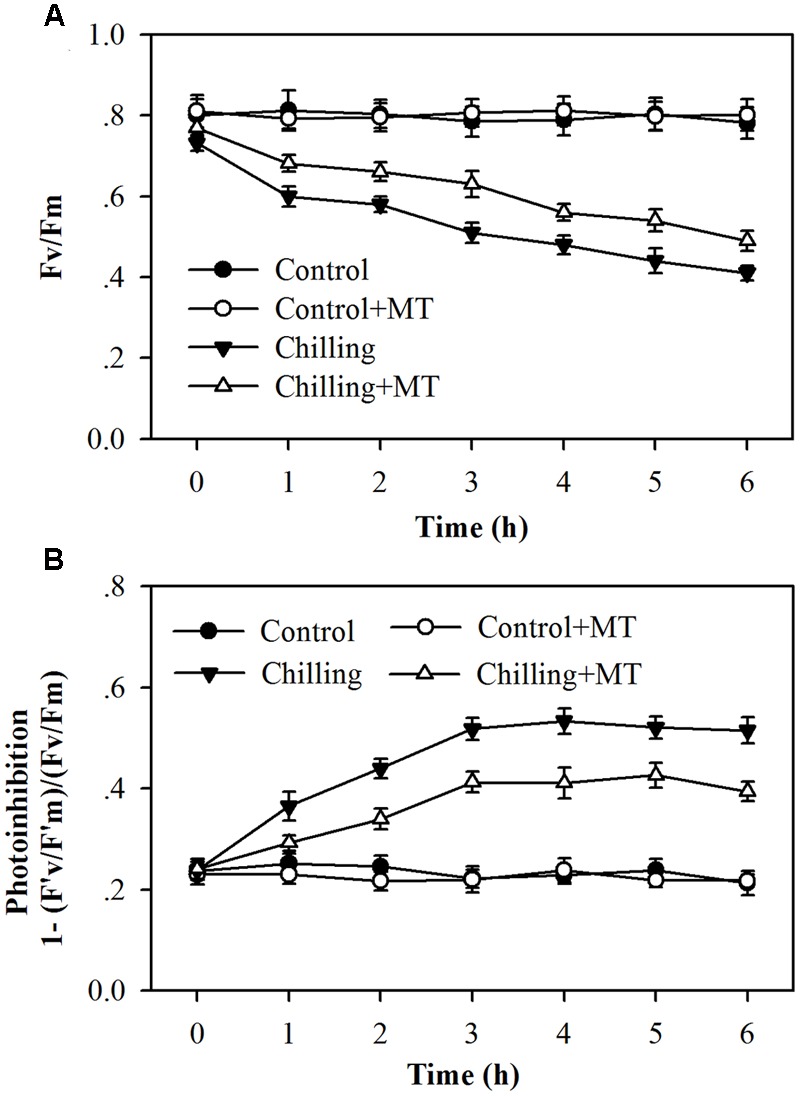
**Changes in photoinhibition in the leaves of tomato seedlings pretreated with melatonin (MT) following exposure to light during chilling. (A)** The maximum quantum yield (Fv/Fm) of PSII; **(B)** Photoinhibition of PSII. Leaves of tomato (*Solanum lycopersicum* L. cv. Micro-Tom) seedlings at the three-leaf stage were pretreated with 100 μmol melatonin (MT) one time a day for 3 days. At the end of light cycle at 18:00 on day 3, seedlings were exposed to chilling (4°C) for 10 h in the dark, then in the light (400 μmol m^-2^ s^-1^) next morning for another 6 h. Data were collected at 0, 1, 2, 3, 4, 5, and 6 h following light exposure. The values presented are mean ± SD (*n* = 6).

Tomato plants have been demonstrated to undergo severe photoinhibition under high light or low light in combination with low temperatures (Zhang and Scheller, 2004; Han et al., 2010; Huang et al., 2010). To investigate the effects of exogenous melatonin on photoinhibition in tomato seedlings exposed to moderate light during chilling, we measured the effective photochemical efficiency (F′v/F′m) and the maximum quantum yield (Fv/Fm) of PSII. The photoinhibition was estimated by calculation of 1–(F′v/F′m)/(Fv/Fm). It was found that chilling (4°C) in the dark for 10 h did not cause significant reductions in Fv/Fm and F′v/F′m (at time 0), however, chilling in the light (400 μmol m^-2^ s^-1^) dramatically decreased Fv/Fm and F′v/F′m in tomato seedlings (**Figure 1A**; Supplementary Figure S1). It is notable that higher F′v/F′m and Fv/Fm were observed in melatonin-pretreated seedlings than in non-melatonin-treated ones under chilling and moderate light conditions, showing reduced photoinhibition in melatonin-treated seedlings (**Figure 1B**). These results indicate that exogenous application of melatonin alleviates photoinhibition in tomato seedlings exposed to chilling and light.

### Melatonin Accelerates Non-photochemical Quenching in Tomato Seedlings Exposed to Moderate Light during Chilling

**FIGURE 2:**
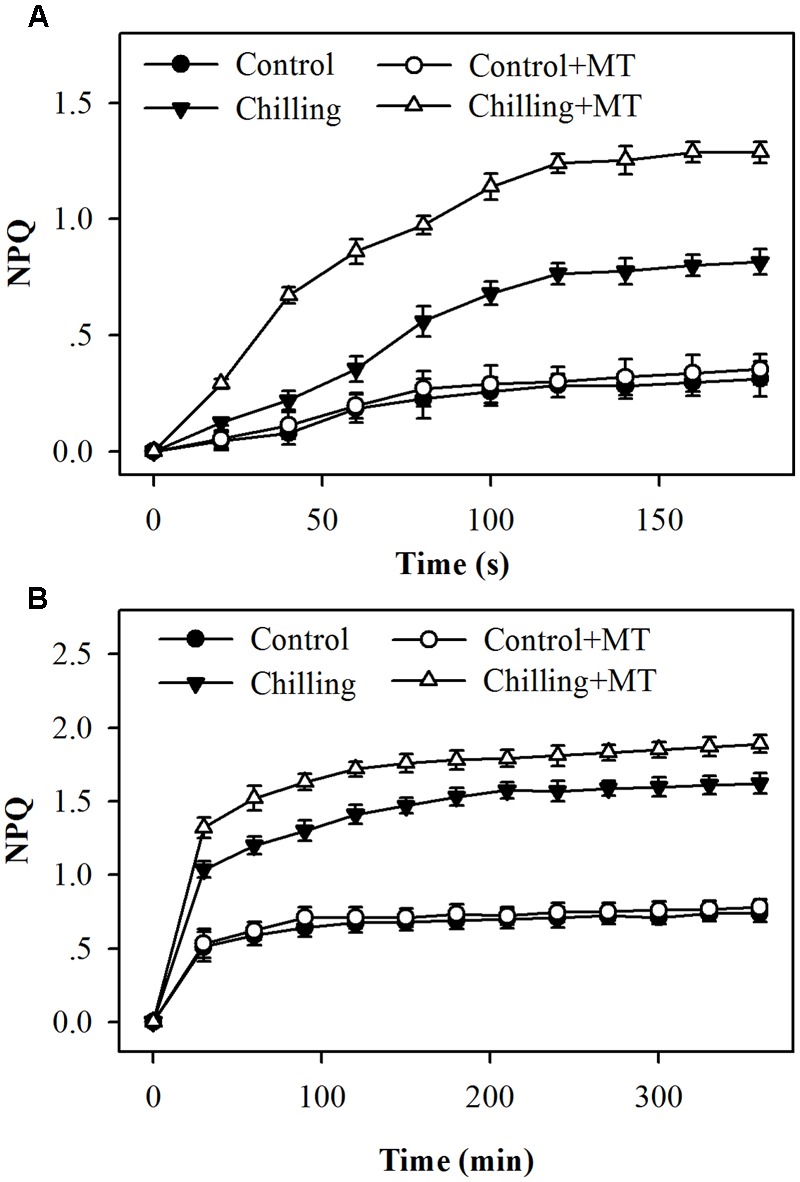
**Kinetics of NPQ induction following exposure to light for (A)** the initial 180 s or **(B)** 360 min in the leaves of tomato seedlings pretreated with melatonin (MT) during chilling. Leaves of tomato (*Solanum lycopersicum* L. cv. Micro-Tom) seedlings at the three-leaf stage were pretreated with 100 μmol melatonin (MT) one time a day for 3 days. At the end of light cycle at 18:00 on day 3, seedlings were exposed to chilling (4°C) for 10 h in the dark, then in the light (400 μmol m^-2^ s^-1^) next morning for another 6 h. Data were collected at 20 s intervals for the initial 180 s and then every 30 min for 6 h following light exposure. The values presented are mean ± SD (*n* = 6).

We examined whether NPQ contributed to reduced photoinhibition observed in the first experiment and whether melatonin treatment affected NPQ in tomato seedlings under chilling and light conditions. The assessment of NPQ showed that in response to light during chilling, NPQ was induced rapidly within as short as 20 s, and seedlings pretreated with melatonin exhibited a faster and higher induction of NPQ than seedlings without melatonin application (**Figure 2A**). Following 20 s of exposure to light during chilling, melatonin-pretreated seedlings showed a 53% increase in NPQ in comparison with non-melatonin-treated seedlings (**Figure 2A**). Over a course of 6 h, the levels of NPQ in melatonin-treated seedlings remained significantly higher than those in non-melatonin-treated seedlings under chilling and light conditions (**Figure 2B**). These results indicate that melatonin increases the initial induction and final level of NPQ under moderate light during chilling.

### Melatonin Promotes De-epoxidation of Xanthophyll

**FIGURE 3:**
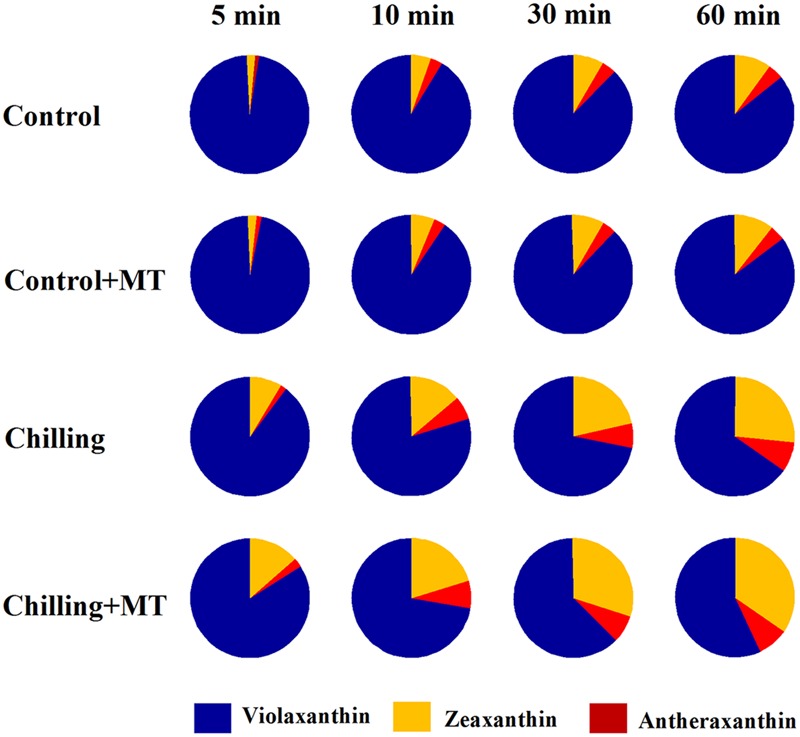
**Alteration of violaxanthin de-epoxidation in the leaves of tomato seedlings pretreated with melatonin (MT) following exposure to light during chilling.** Leaves of tomato (*Solanum lycopersicum* L. cv. Micro-Tom) seedlings at the three-leaf stage were pretreated with 100 μmol melatonin (MT) one time a day for 3 days. At the end of light cycle at 18:00 on day 3, seedlings were exposed to chilling (4°C) for 10 h in the dark, then in the light (400 μmol m^-2^ s^-1^) next morning for another 6 h. Samples were collected at 0, 5, 10, 30, and 60 min following light exposure and xanthophyll pigments were quantitated by HPLC. The values presented are mean ± SD (*n* = 3).

**FIGURE 4:**
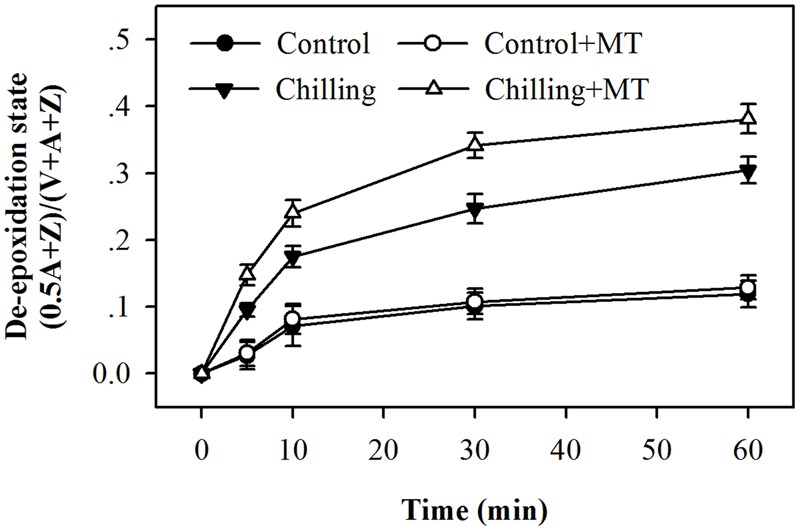
**Changes in the de-epoxidation state of xanthophyll pigments in the leaves of tomato seedlings pretreated with melatonin (MT) following exposure to light during chilling.** Leaves of tomato (*Solanum lycopersicum* L. cv. Micro-Tom) seedlings at the three-leaf stage were pretreated with 100 μmol melatonin (MT) one time a day for 3 days. At the end of light cycle at 18:00 on day 3, seedlings were exposed to chilling (4°C) for 10 h in the dark, then in the light (400 μmol m^-2^ s^-1^) next morning for another 6 h. Samples were collected at 0, 5, 10, 30, and 60 min following light exposure and xanthophyll pigments were quantitated by HPLC. The values presented are mean ± SD (*n* = 3).

In order to investigate the possible mechanism of melatonin-mediated increase in NPQ under chilling and light conditions, we determined the effects of melatonin treatment on the xanthophyll cycle, which has been proved to contribute substantially to NPQ (Holt et al., 2004). The de-epoxidation state of the xanthophyll pigments was examined in tomato seedlings following exposure to light under chilling stress. Violaxanthin predominated in fully dark-adapted seedlings whereas antheraxanthin and zeaxanthin were generated rapidly in response to light during chilling. Higher levels of antheraxanthin and zeaxanthin were observed in melatonin-pretreated seedlings than in non-melatonin-pretreated ones (**Figure 3**) under moderate light in combination with chilling. In order to determine whether the rapid induction of NPQ in melatonin-treated seedlings were due to the increased de-epoxidation of violaxanthin, we measured the extent of de-epoxidation in seedlings pretreated either with or without melatonin following exposure to light under chilling condition. Following exposure to 400 μmol m^-2^ s^-1^ light during chilling, a significant increase in de-epoxidation of violaxanthin to zeaxanthin was observed in melatonin-treated seedlings within 5 min, with additional de-epoxidation occurring upon longer exposure to light (**Figure 4**). In contrast, the rate of de-epoxidation was lower in seedlings without melatonin application, resulting in a lower de-epoxidation state. These results indicate that exogenous melatonin promoted de-epoxidation activity in tomato seedlings under moderate light during chilling, consistent with the rapid initial induction of NPQ.

### Melatonin Induces *VDE* Expression and Increases VDE Activity

**FIGURE 5:**
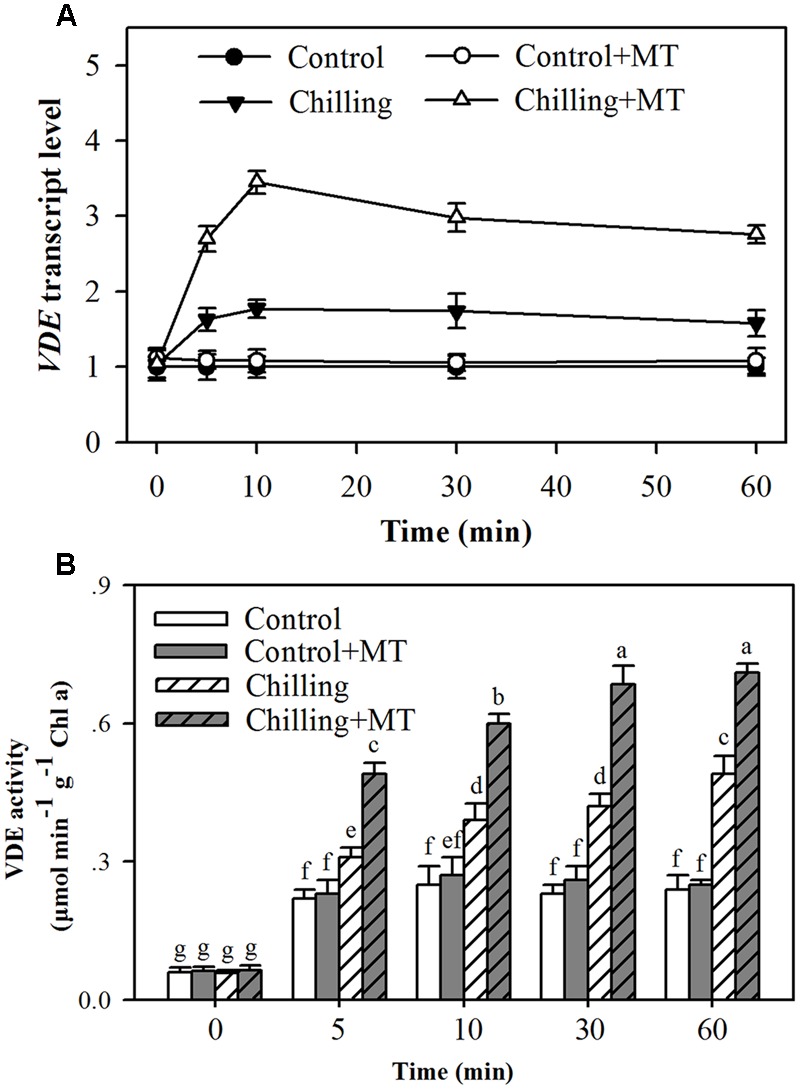
**Effects of melatonin on VDE gene expression and VDE activity in the leaves of tomato seedlings following exposure to light during chilling. (A)** *VDE* transcript level; **(B)** VDE activity. Leaves of tomato (*Solanum lycopersicum* L. cv. Micro-Tom) seedlings at the three-leaf stage were pretreated with 100 μmol melatonin (MT) one time a day for 3 days. At the end of light cycle at 18:00 on day 3, seedlings were exposed to chilling (4°C) for 10 h in the dark, then in the light (400 μmol m^-2^ s^-1^) next morning for another 6 h. Samples were collected at 0, 5, 10, 30, and 60 min following light exposure. The values presented are mean ± SD (*n* = 3). Different letters indicate significant differences at *P* < 0.05 among treatments.

The conversion of violaxanthin to zeaxanthin and antheraxanthin in the xanthophyll cycle depends on light-activated VDE. The increase in de-epoxidation activity observed in melatonin-treated tomato seedlings could result from an increase in the expression of *VDE* mRNA or (and) activation of VDE activity. Therefore, to further investigate the impacts of melatonin on the de-epoxidation of violaxanthin in the xanthophyll cycle, we measured *VDE* transcript abundance and VDE activity in tomato seedlings exposed to light in combination with chilling. Higher *VDE* expression was observed in melatonin-treated seedlings than in non-melatonin-treated ones under chilling and light conditions (**Figure 5A**). Moreover, exogenous application of melatonin led to the highest transcript level 10 min following illumination during chilling (**Figure 5A**). VDE activation requires the light-mediated acidification of the thylakoid lumen where VDE resides. The results showed that VDE activity was significantly increased by moderate light during chilling and the increase was much greater in melatonin-treated seedlings than in non-melatonin-treated seedlings and control seedlings (**Figure 5B**). The highest VDE activity was observed 60 min following light exposure under chilling condition (**Figure 5B**). These results suggest that melatonin promotes *VDE* expression and stimulates VDE activity.

### Effects of Melatonin on Xanthophyll De-epoxidation are Counteracted by Feeding Dithiothreitol

**FIGURE 6:**
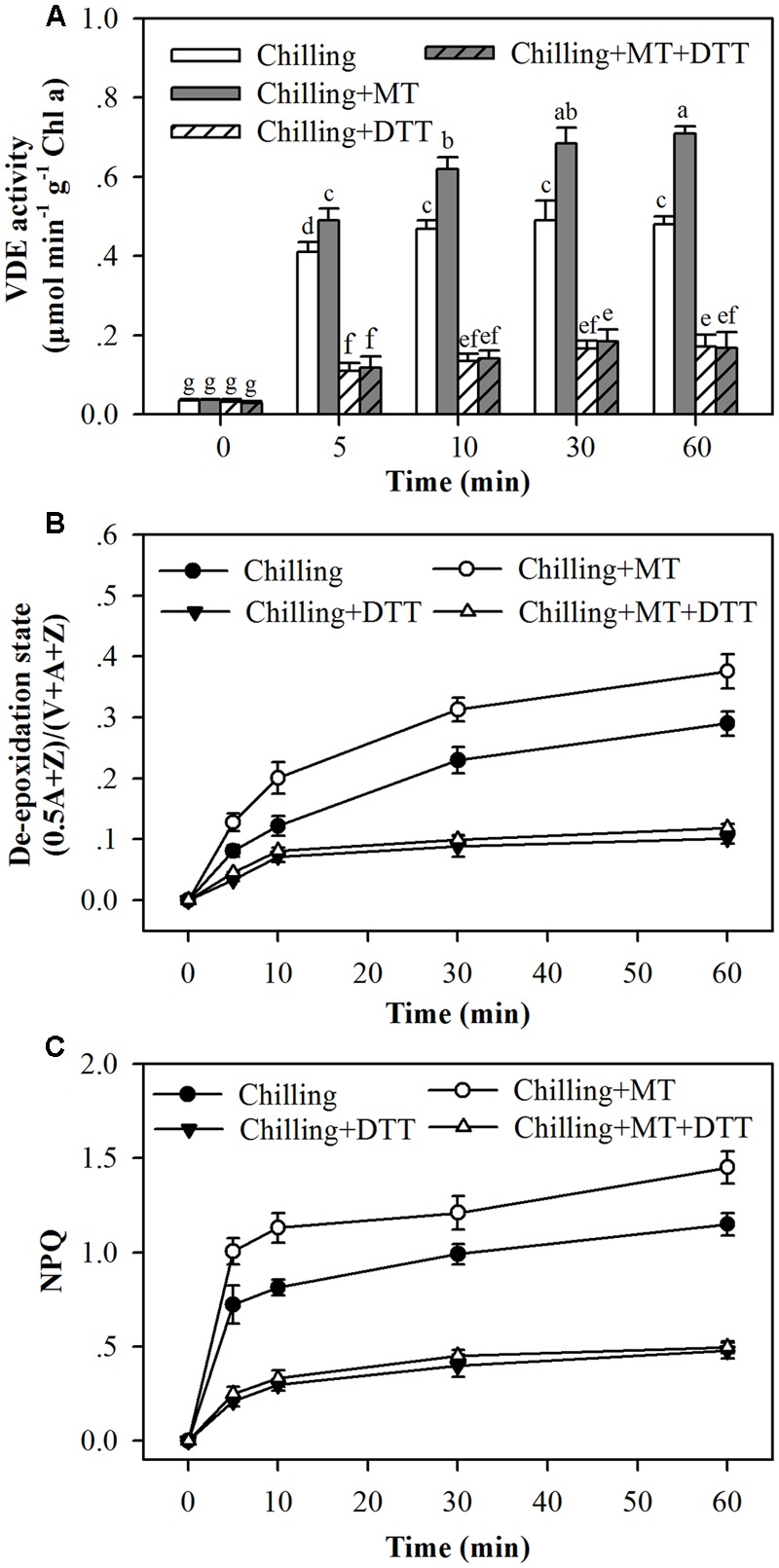
**Dithiothreitol blocked the effects of melatonin on (A)** VDE activity, **(B**) the de-epoxidation state of xanthophyll pigments and **(C)** the induction of NPQ in the leaves of tomato seedlings following exposure to light during chilling. Leaves of tomato (*Solanum lycopersicum* L. cv. Micro-Tom) seedlings at the three-leaf stage were pretreated with 100 μmol melatonin (MT) one time a day for 3 days. At the end of light cycle at 18:00 on day 3, they were exposed to chilling (4°C) for 10 h in the dark, then in the light (400 μmol m^-2^ s^-1^) next morning for another 6 h. Tomato seedlings were infiltrated with either 5 mM dithiothreitol (DTT) or with water from stem 2 h before they were exposed to light. Samples were collected at 0, 5, 10, 30, and 60 min following light exposure. The values presented are mean ± SD (*n* = 3). Different letters indicate significant differences at *P* < 0.05 among treatments.

Dithiothreitol is an inhibitor of VDE (Yamamoto and Komite, 1972). To further ascertain the role of melatonin in promoting de-epoxidation of violaxanthin and NPQ, tomato seedlings pretreated with or without melatonin were fed with DTT. Feeding seedlings with DTT suppressed VDE activities in all examined seedlings and eliminated the effects of melatonin on the de-epoxidation state of the xanthophyll cycle (**Figures 6A,B**). Furthermore, application of DTT dramatically suppressed the development of NPQ (**Figure 6C**). These results suggest that melatonin regulates the xanthophyll cycle and NPQ by mainly acting on VDE activity.

### Melatonin Increases *DHAR* Expression, DHAR Activity and the Level of Ascorbic Acid

**FIGURE 7:**
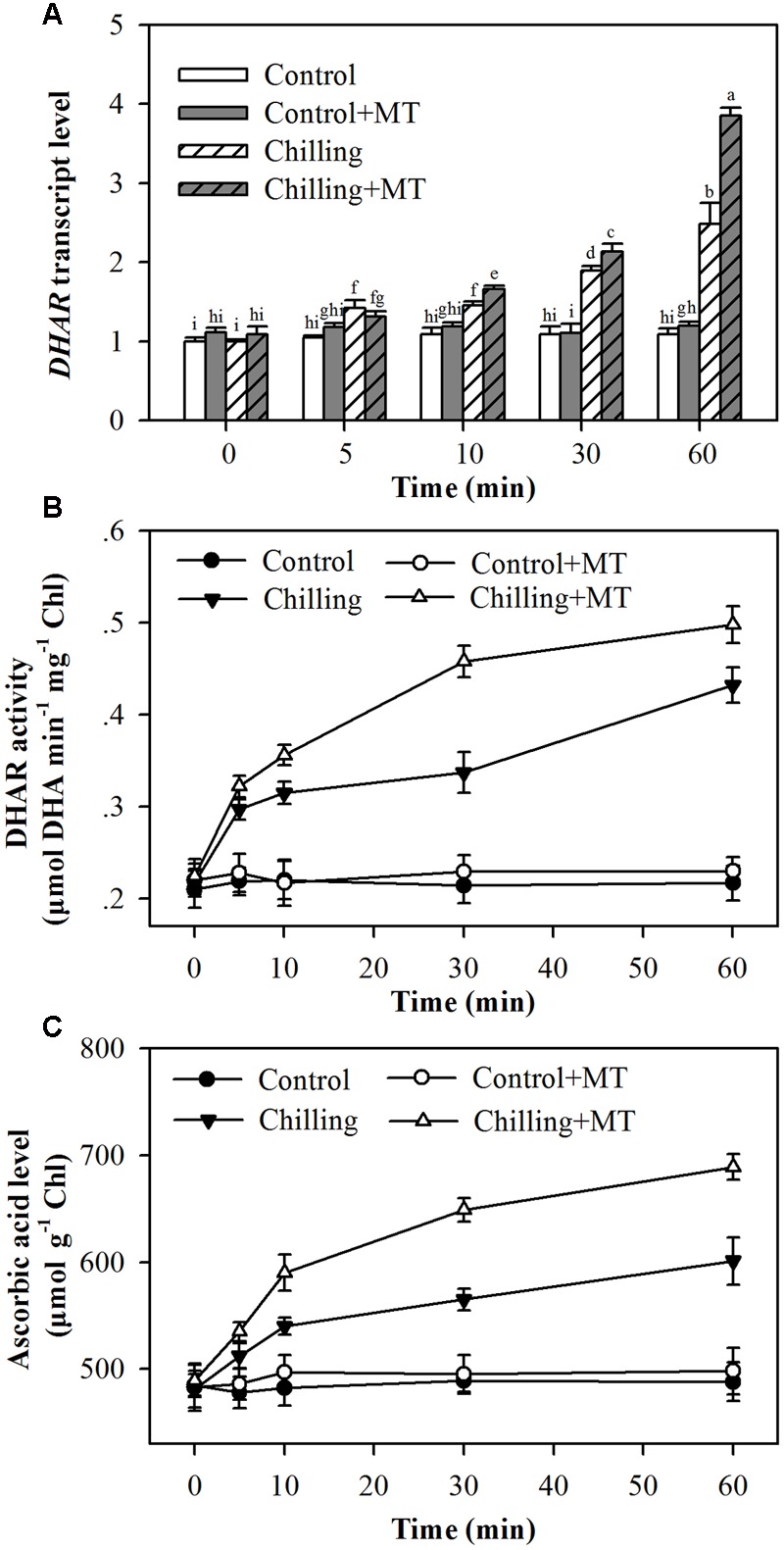
**Effects of melatonin on (A)** *DHAR* transcript level, **(B)** DHAR activity and **(C)** ascorbic acid level in the leaves of tomato seedlings following exposure to light during chilling. Leaves of tomato (*Solanum lycopersicum* L. cv. Micro-Tom) seedlings at the three-leaf stage were pretreated with 100 μmol melatonin (MT) one time a day for 3 days. At the end of light cycle at 18:00 on day 3, seedling were exposed to chilling (4°C) for 10 h in the dark, then in the light (400 μmol m^-2^ s^-1^) next morning for another 6 h. Samples were collected at 0, 5, 10, 30, and 60 min following light exposure. The values presented are mean ± SD (*n* = 3). Different letters indicate significant differences at *P* < 0.05 among treatments.

To catalyze the de-epoxidation reaction, VDE requires ascorbic acid as a cofactor. It has been demonstrated that increased *DHAR* expression and ascorbic acid content mitigate photoinhibition by improving VDE activity in tobacco plants (Chen and Gallie, 2008). To determine the possible mechanism of melatonin-mediated increase in VDE activity, we measured transcript abundance of *DHAR*, DHAR activity and level of ascorbic acid in the chloroplasts of tomato seedlings subject to chilling and moderate light. It was observed that expression of *DHAR*, DHAR activity and level of ascorbic acid were increased by chilling and the increase was much greater when exogenous melatonin was applied in tomato seedlings (**Figures 7A,B**). Together with melatonin-mediated increase in VDE activity, these results may suggest that melatonin-mediated increase in the level of ascorbic acid contributes, at least in part, to the increased VDE activity in melatonin-treated tomato seedlings exposed to light under chilling.

## Discussion

Non-photochemical quenching is an important physiological process employed by plants to dissipate excess absorbed light energy. NPQ is induced when absorbed light surpasses the capacity of light utilization in photochemistry, which generally occurs under conditions of high light or low light in combination with other abiotic stresses (Demmig-Adams, 1990; Huang et al., 2010). A substantial part of NPQ is attributed to the xanthophyll cycle, in which zeaxanthin is generated in a light-dependent manner (Eskling et al., 1997). In the present study, we have concluded that melatonin, an extensively studied molecule in plants, protects tomato seedlings against photoinhibition under moderate light during chilling. The evidence leading to the conclusion includes (1) application of melatonin accelerates NPQ by increasing rates of VDE; (2) melatonin-mediated increase in NPQ is a consequence of elevated VDE activity; and (3) increased VDE activity is due to melatonin-induced expression of *VDE* and melatonin-mediated accumulation of VDE cofactor ascorbic acid.

Melatonin plays a recognized role in the protection of plants against various abiotic stresses. There are several reports on the application of melatonin and its influence on photosynthetic apparatus under stress conditions (Fan et al., 2015; Szafrańska et al., 2016; Zhao et al., 2016; Zhou et al., 2016). These studies have established that melatonin gives an advantage to the function of photosystems by reducing oxidative damages through scavenging of ROS or regulation of electron transport chain. However, information regarding the effects of melatonin on the xanthophyll cycle and the induced NPQ is still lacking in plants. It was observed in this study that moderate light during chilling greatly inhibited PSII, whereas melatonin application significantly alleviated this inhibition, suggesting a protective role of melatonin in amelioration of photo damage. Though the widely reported role of melatonin in promoting the capacity of scavenging ROS may contribute to the alleviated inhibition of PSII in this study, yet there might be an alternative mechanism. In order to pursue additional mechanism, we assessed the impacts of melatonin on NPQ in tomato seedlings exposed to light under chilling stress, because NPQ is indispensable to the dissipation of excess light absorbed in photosystem and thus confers protection of PSII against photoinhibition. In this study, NPQ was rapidly induced in response to moderate light during chilling in dark-adapted tomato seedlings, showing that 400 μmol m^-2^ s^-1^ is excessive in tomato seedlings subject to chilling (**Figure 2**). This observation is consistent with a previous study that low temperatures combined with light increase NPQ as a mechanism of dissipating excess energy as heat (Corcuera et al., 2005). The chilling-light induction of NPQ was further enhanced by the application of melatonin, supporting that melatonin is beneficial in accelerating diversion of absorbed light from photochemistry under chilling condition. However, it is unclear based on the data presented here that to what extent melatonin-mediated increases in the NPQ levels contribute to relieved photoinhibition, because melatonin is a molecule functioning at multiple levels in plants. Melatonin can serve as direct scavenger of ROS and it also promotes the expression of antioxidant enzymes and enhances the accumulation of antioxidants, thus leading to reduced level of ROS, which may be partially accountable for the alleviated photoinhibition in this study. Therefore, in future studies, it is worth comparing the role of melatonin-mediated increases in NPQ with that of melatonin-mediated reductions of ROS in the alleviation of photoinhibition.

In agreement with the increased induction of NPQ by melatonin was the observed rise in de-epoxidation state of violaxanthin in the xanthophyll cycle. Melatonin significantly increased the conversion of violaxanthin to antheraxanthin and zeaxanthin after dark-adapted tomato seedlings were exposed to light during chilling. Kinetics of xanthophyll de-epoxidation in seedlings showed that melatonin accelerated the rate of de-epoxidation and maintained a high level of de-epoxidation state under moderate light and chilling condition (**Figure 4**). Our results further support the previously established notion that the formation of NPQ upon either excess light or low light combined with other stresses matches the changes in de-epoxidation state of xanthophyll (Johnson et al., 2008; Ware et al., 2015). The de-epoxidation of xanthophyll is catalyzed by VDE, which is a central player in the xanthophyll cycle. A previous study has confirmed that chilling leads to reduction in VDE activity, thus resulting in lower rate of de-epoxidation and retarded formation of NPQ (Chen and Gallie, 2012). Thus, the observed increase in de-epoxidation state due to melatonin application in our study is supposed to be in line with higher activity of VDE. It was shown that VDE activity was higher in melatonin-treated seedlings than in non-melatonin-treated ones under chilling and light conditions, which substantiates that melatonin increased larger de-epoxidation state of xanthophyll and induced greater NPQ by acting on VDE activity.

Enzyme activity can be influenced by several factors, including transcript levels, protein turnover and cofactors. *VDE* transcript levels increased in response to light during chilling, and application of melatonin resulted in a dramatic increase in *VDE* transcript level. Overall, melatonin-mediated increase in transcript levels appeared consistent with the increase in VDE activity, indicating that increased *VDE* expression induced by melatonin contributes to enhanced VDE activity. Transcript level, however, did not always match VDE activity in the presented results. Peak transcript level occurred 10 min following illumination during chilling, while peak VDE activity was observed at 60 min (**Figure 5A**). The difference in transcript level and VDE activity may demonstrate that this enzyme does not turn over rapidly and this result is in accordance with a previous study (Bugos et al., 1999). To have catalytic activity, VDE also requires the presence of ascorbic acid, which is believed to function as a cofactor (Bratt et al., 1995); we therefore ask if melatonin-mediated increase in VDE activity is associated with the regulation of ascorbic acid generation in tomato seedlings. In this study, melatonin-pretreated seedlings accumulated more ascorbic acid than non-melatonin-pretreated ones did under chilling stress. Moreover, melatonin application significantly promoted the expression of DHAR, which is responsible for the production of ascorbic acid in plants. It was also found that melatonin enhanced DHAR activity in tomato seedlings (**Figure 7**). In fact, it has been firmly established that melatonin is in favor of ascorbic acid production in plants under various stress conditions (Li et al., 2012; Fan et al., 2015; Shi et al., 2015a). These lines of evidence support that melatonin stimulates VDE activity, at least in part, by promoting VDE expression and accumulation of VDE cofactor ascorbic acid.

Evidence presented in this study supports that melatonin promotes NPQ by acting on VDE activity. It is thus can be speculated that inhibition of VDE activity would lead to decreased de-epoxidation state of xanthophyll and reduced levels of NPQ. Thus, in order to inhibit VDE activity, tomato seedlings were fed with DTT, a well-known VDE inhibitor. VDE was inactivated by DTT in both melatonin-treated seedlings and non-melatonin-treated ones under light and chilling conditions. In addition, the de-epoxidation of xanthophyll was inhibited no matter whether or not melatonin was applied. It was also the case for NPQ as a consequence of inhibited de-epoxidation of xanthophyll (**Figure 6**). These results showed that the effects of melatonin on NPQ were eliminated by addition of DTT, further demonstrating that melatonin-mediated regulation of NPQ is achieved through the control of de-epoxidation of xanthophyll, which is ultimately regulated by melatonin-mediated changes in VDE activity.

In summary, we have found that exogenous application of melatonin alleviated photoinhibition in tomato seedlings exposed to moderate light during chilling. The possible mechanism is that melatonin-mediated increases in *VDE* transcript level and ascorbic acid level contribute to higher VDE activity in tomato seedlings exposed to light during chilling, resulting in an increase in the de-epoxidation state of xanthophyll cycle and the induction of NPQ. Relieved photoinhibition is, at least in part, attributed to higher NPQ in melatonin-pretreated tomato seedlings exposed to moderate light during chilling.

## Author Contributions

FD, MW, and SZ designed the study. FD, MW, and BL performed the experiments and analyzed the data. FD wrote the manuscript. MW and SZ revised the manuscript.

## Conflict of Interest Statement

The authors declare that the research was conducted in the absence of any commercial or financial relationships that could be construed as a potential conflict of interest.
