# Supplementary material for: Exogenous Melatonin Mitigates Photoinhibition by Accelerating Non-photochemical Quenching in Tomato Seedlings Exposed to Moderate Light during Chilling
Source: Front Plant Sci. 2017 Feb 20;8:244. doi: 10.3389/fpls.2017.00244 (PMC5316535; doi:10.3389/fpls.2017.00244)
Supplement: Supplementary file 1 [file Data_Sheet_1.PDF]

Supplementary Material

Exogenous melatonin mitigates photoinhibition by accelerating non-photochemical quenching in tomato seedlings exposed to moderate light during chilling

Fei Ding[1,†], Meiling Wang[2,†], Bin Liu[1], Shuoxin Zhang[1*]

[*]**Correspondence:** Shuoxin Zhang sxzhang@nwsuaf.edu.cn

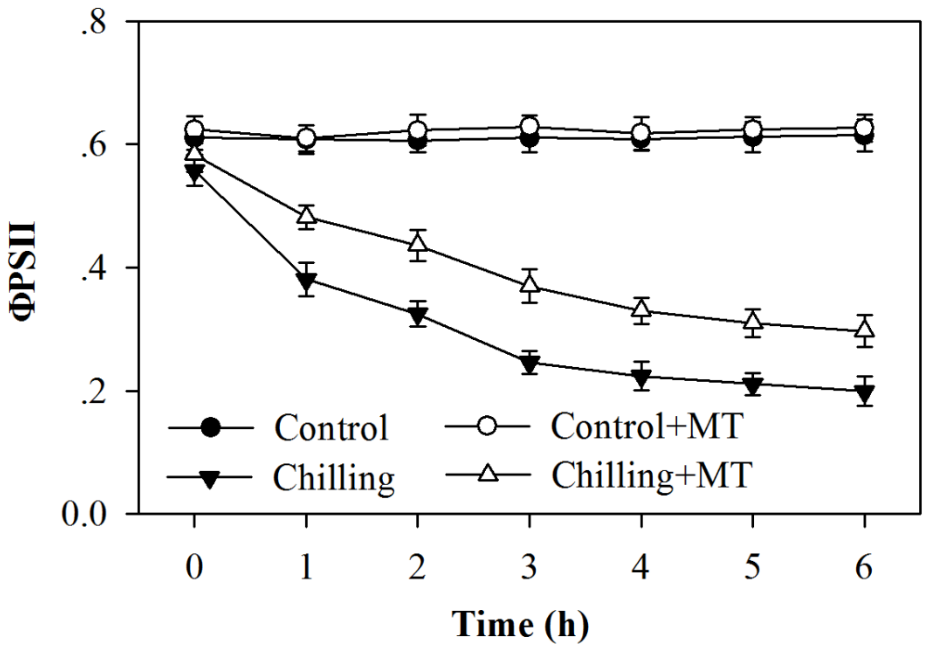

Supplementary Figure 1. Changes in the effective photochemical efficiency ΦPSII (F′v/F′m) of PSII in the leaves of tomato seedlings pretreated with melatonin (MT) following exposure to light during chilling. Leaves of tomato (*Solanum lycopersicum* L. cv. Micro-Tom) seedlings at the three-leaf stage were pretreated with 100 µmol melatonin (MT) one time a day for 3 days. At the end of light cycle at 18:00 on day 3, seedlings were exposed to chilling (4 ℃) for 10 h in the dark, then in the light (400 µmol m^{-2} s^{-1}) next morning for another 6 h. Data were collected at 0, 1, 2, 3, 4, 5 and 6 h following light exposure. The values presented are means ± SDs (n = 6).
